# Role of MicroRNA-204 in Regulating the Hallmarks of Breast Cancer: An Update

**DOI:** 10.3390/cancers16162814

**Published:** 2024-08-10

**Authors:** Mercedes Bermúdez, Marcela Guadalupe Martínez-Barajas, Lesly Jazmín Bueno-Urquiza, Jorge Armando López-Gutiérrez, Carlos Esteban Villegas-Mercado, César López-Camarillo

**Affiliations:** 1 Faculty of Dentistry, Autonomous University of Chihuahua, Chihuahua 31000, Mexico; cmercado@uach.mx; 2 University Center for Health Sciences, University of Guadalajara, Guadalajara 44340, Mexico; marcela.martinez8631@alumnos.udg.mx (M.G.M.-B.); lesly.bueno@alumnos.udg.mx (L.J.B.-U.); 3 Faculty of Dentistry, Autonomous University of Sinaloa, Josefa Ortiz de Domínguez s/n y Avenida de las Américas, Culiacan 80013, Mexico; doctorjorgelopez@uas.edu.mx; 4 Genomic Sciences Program, Autonomous University of Mexico City, San Lorenzo 290, Col del Valle, Mexico City 03100, Mexico

**Keywords:** miR-204, microRNAs, breast cancer, hallmarks of cancer

## Abstract

**Simple Summary:**

This review aims to explore the role of the microRNA-204 (miR-204), a tumor suppressor gene, in the development of breast cancer, focusing on how it influences the hallmarks of cancer. By understanding the impact of miR-204 on these hallmarks, this review seeks to shed light on its potential as a molecular target for breast cancer therapy.

**Abstract:**

microRNA-204-5p (miR-204) is a small noncoding RNA with diverse regulatory roles in breast cancer (BC) development and progression. miR-204 is implicated in the instauration of fundamental traits acquired during the multistep development of BC, known as the hallmarks of cancer. It may act as a potent tumor suppressor by inhibiting key cellular processes like angiogenesis, vasculogenic mimicry, invasion, migration, and metastasis. It achieves this by targeting multiple master genes involved in these processes, including HIF-1α, β-catenin, VEGFA, TGFBR2, FAK, FOXA1, among others. Additionally, miR-204 modulates signaling pathways like PI3K/AKT and interacts with HOTAIR and DSCAM-AS1 lncRNAs, further influencing tumor progression. Beyond its direct effects on tumor cells, miR-204 shapes the tumor microenvironment by regulating immune cell infiltration, suppressing pro-tumorigenic cytokine production, and potentially influencing immunotherapy response. Moreover, miR-204 plays a crucial role in metabolic reprogramming by directly suppressing metabolic genes within tumor cells, indirectly affecting metabolism through exosome signaling, and remodeling metabolic flux within the tumor microenvironment. This review aims to present an update on the current knowledge regarding the role of miR-204 in the hallmarks of BC. In conclusion, miR-204 is a potential therapeutic target and prognostic marker in BC, emphasizing the need for further research to fully elucidate its complex roles in orchestrating aggressive BC behavior.

## 1. Introduction

Cancer is a disease characterized by uncontrolled proliferation by transformed cells subject to evolution by natural selection, ultimately leading these cells to a lethal phenotype [1] that can emerge in almost any part of the body, with breast cancer (BC) being the most commonly diagnosed type and the primary cause of cancer-related deaths among women globally [2,3]. Approximately 2.3 million new cases of BC were reported in 2020, accounting for one-fourth of all new cancer cases, leading to around 685,000 deaths, representing one-sixth of all cancer-related deaths that year [3,4].

BC is a diverse disease from a clinical standpoint. Estrogen receptor (ER) expression plays a significant role in classifying ER-expressing (ER^+^), and non-ER-expressing (ER^−^) tumors [5]. Additionally, BC can be categorized as basal-like or non-basal-like, according to the cell type of origin; luminal or basal/myoepithelial cells. The “triple-negative” subtype, which constitutes around 10% of all BCs, lacks estrogen receptor (ER), progesterone receptor (PR), and human growth factor/neu receptors (Her2) while exhibiting high levels of basal cytokeratins (K5-K14) [6]. Non-basal-like cancer further divides into subtypes such as luminal A (ER^high^/Her2^low^), luminal B (ER^low^/Her2^low^), and Her2-enriched (ER^−^, Her2^+^) [6].

Identifying the specific type of BC is essential for enhancing clinical results, as there are notable variations in biology and how they respond to treatment among these types [7]. This heterogeneity frequently leads to wrong diagnosis and thus an incorrect treatment favoring its evolution and further metastasis, resistance to treatment [8], and leading to an elevated rate of mortality in BC patients [9]. Thus, understanding the varied causes behind these different subtypes will aid in treatment guidance, survival rate prediction, and the development of prevention strategies due to their intricate biology [10].

Numerous factors have been linked to the development of BC, including alcohol intake, body mass index, height, mammographic density, age at first menstruation (menarche), menopausal status, physical activity, smoking, and type 2 diabetes mellitus (T2DM) [11]. Nevertheless, its pathophysiology is not completely understood given the complexity of molecular networks that participate in the development of the disease where coding and non-coding genes are involved.

microRNAs (miRNAs) are small non-coding RNAs typically from 18 to 25 nucleotides in length [12,13,14]; they play a role in regulating post-transcriptional gene expression and have been recognized for their involvement in controlling cell differentiation, proliferation, and survival under both normal and pathological circumstances [15]. miRNAs regulate approximately 30% of genes at the post-transcriptional level [16]. They inhibit the translation and stability of messenger RNAs (mRNAs) and influence genes related to various cellular processes such as inflammation, cell cycle regulation, stress response, differentiation, apoptosis, and migration [16]. Recent studies indicate that mutations or interference with miRNAs are associated with various types of human cancers, implying that miRNAs may serve as either tumor suppressors or oncogenes [17]. Tumor suppressor miRNAs are frequently absent in cancer, typically due to various mechanisms such as promoter methylation, mutations or deletions, or problems with miRNA processing [18].

**Figure 1:**
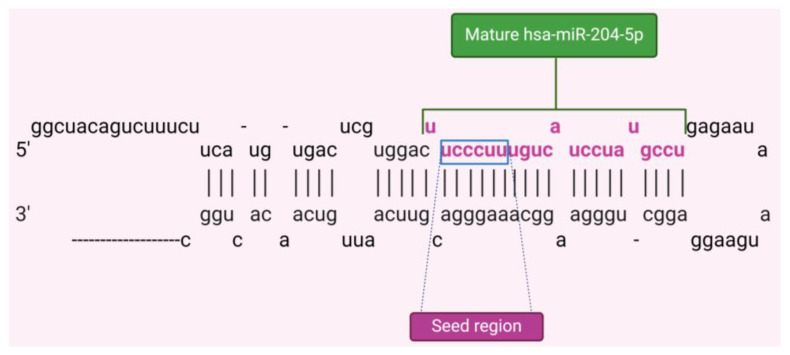
Stem-loop pre-miRNA structure of hsa-miR-204-5p with mature miR-204 sequence in pink and seed region in the blue box.

miRNA-204-5p (miR-204) is a highly tissue-specific microRNA that plays a crucial role in regulating molecular systems and pathophysiological conditions [19]. Its abundance is controlled by various transcriptional and post-transcriptional mechanisms, impacting disease processes by targeting multiple biological pathways in specific tissues. The gene for miR-204, MIR204, is in intron 9 of the TRPM3 gene on the long arm of human chromosome 9; this gene encodes a pre-miR-204 stem-loop that is transcribed in the same direction as its host gene (Figure 1) [20]. In terms of molecular regulation, miR-204 expression can be modulated by DNA methylation and transcription factors like Pax6 and STAT3 [21,22]. DNA methylation in the promoter CpG island of the TRPM3/miR-204 gene can lead to downregulation observed in certain cancers while activated STAT3 can bind to regulatory regions of TRPM3, reducing miR-204 expression [20]. miR-204 is considered a tumor suppressor that could regulate relevant processes like tumor growth and metastasis, and the remodeling of the immune microenvironment in BC [23].

There is evidence that miR-204 is involved in the instauration of fundamental traits acquired during the multistep development of human tumors, known as the hallmarks of cancer. According to Hanahan, the hallmarks of cancer include sustaining proliferative signaling, evading growth suppressors, resisting cell death, enabling replicative immortality, inducing angiogenesis, and activating invasion and metastasis; additionally, emerging hallmarks and enabling characteristics have been recognized as comprising deregulating cellular energetics, avoiding immune destruction, genome instability and mutation, tumor-promoting inflammation, nonmutational epigenetic reprogramming, polymorphic microbiomes, senescent cells, and unlocking phenotype plasticity [24]. These hallmarks provide a useful conceptual framework for understanding the complexity of cancer biology; thus, this review aims to present current knowledge regarding the role of miR-204 in the hallmarks of BC.

## 2. Effects of the Abnormal Expression of miR-204 in Human Cancers

Understanding the modulation functions of this miRNA is complicated, since the findings differ due to cellular heterogeneity in different types of cancers. It has been shown that miR-204 plays a tumor inhibitory role and its downregulation promotes tumorigenesis in ovarian cancer (OC), prostate cancer (PC), and BC, and DNA methylation epigenetically silences miR-204 in papillary thyroid carcinoma and colorectal cancer (CRC) cells [25,26]. Nevertheless, a limited number of studies demonstrate that miR 204 is overexpressed in OC, PC, and BC [23,27,28,29,30,31,32].

It has been reported that the promoter of the transient receptor potential melastatin 3 (TRPM3) gene is associated with miR-204 and is hypermethylated in CRC and gliomas [26,33]. miR-204 is also known to regulate tumorigenesis and progression by targeting multiple key signaling pathways, as interaction with ncRNAs, including lncRNAs and circRNAs, suppresses the expression or activity of miR-204 in oncocytes [34,35]. Some examples of RNA target coding genes for this miRNA are AKT1, associated with proliferation and metastasis [36], ANGPT1, which promotes angiogenesis [37], and E2F1, which influences the cell cycle [38], among others. Some non-coding RNA targets of miR-204 are NEAT, implicated in proliferation, apoptosis, and the epithelial–mesenchymal transition (EMT) [39,40], SNHG4, involved in radioresistance [41], and PlncRNA-1, which is related to autophagy [42].

There is evidence that a decrease in miR-204 is also found in other solid tumors, such as primary melanomas, gliomas, head and neck tumors, gastric cancer, and endometrial cancer [22,43,44,45]. In non-small-cell lung cancer (NSCLC), different cell lines have been evaluated to elucidate the specific modulation in the progression of this disease, where miR-204 plays a fundamental role with tumorigenic potential; however, this miRNA can suppress NSCLC by attacking ATF2, acting as a tumor suppressor [46]. In lymphoblastic leukemia, it inhibits T-cell proliferation by downregulating SOX4 [47]. It is also known that silencing miR-204 reduces the disease by positively regulating MMP-2 and MMP-9 through NF-κB [48]. In PC, it represses metastasis since it is negatively related to the expression of TRAF1, TAB3, and MAP3K3 and it deactivates signaling via NF-κB [32]. A dual regulatory function of miR-204 is shown in PC, as it is suggested that it is an oncomir by attacking prostate-derived Ets factor (PDEF) and inhibiting tumor suppressor function, but as a tumor suppressor in prostate adenocarcinoma (PAC) cells, LNCaP and 22RV1 for its dual modulatory effects on the expression of key cell cycle regulators, including phosphorylation of AKT, cyclin D1, p21 ^WAF1^, and apoptosis [28].

In the case of BC, the tumor suppressors MX1 and TXNIP have been identified as direct targets of miR-204 [49]. The high association between the expression of miR-204 in BC tissue indicates that miR-204 increases cell proliferation at least in MCF-7 and MDA-MB-231 cells by downregulating tumor suppressor genes [50]. miR-204 is known to regulate the biological behavior of MCF-7 cells by blocking the transcription of FOXA1, a member of the FOX family of transcription factors that is also known as hepatocyte nuclear factor 3α (HNF3α), a potent transcriptional regulator of transthyretin (TTR) and α1-antitrypsin (α1-AT), interacting in lung cancer, thyroid carcinoma, and PC and BC cells, promoting cell growth and inhibiting apoptosis [51]. In a study, the gene expression pattern of miR-204 in 129 breast cancer tissue samples from patients with various subtypes was evaluated. They also analyzed different clinicopathological parameters, including estrogen receptor status, HER-2 status, tumor size, TNM stage, and distant metastasis. The data showed that miR-204 expression was mostly associated with disease progression, with 65 patients having low miR-204 expression and 64 patients having high miR-204 expression. Additionally, the researchers found that miR-204 expression was significantly associated with treatment response, with lower miR-204 levels observed in patients who did not respond favorably to treatment [29]. Overall, the results suggest that miR-204 could be a potential biomarker for prognosis and treatment response in breast cancer patients.

## 3. Roles of miR-204 in the Hallmarks of Breast Cancer

### 3.1. Cell Proliferation

**Figure 2:**
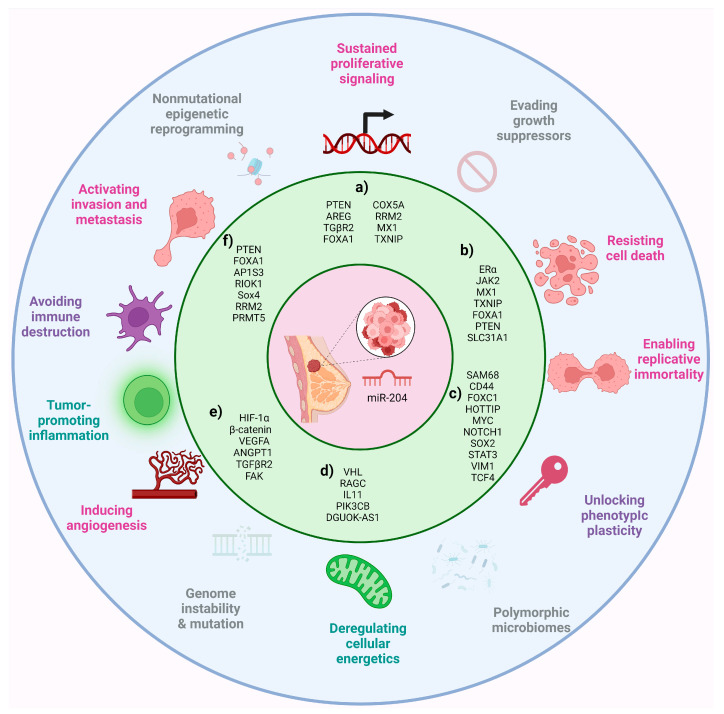
MiR-204 is implicated in the instauration of fundamental traits acquired during the multistep development of BC, known as the hallmarks of cancer. Functional evidence showed that miR-204 is involved in processes including cell proliferation, cell death resistance, chemotherapy resistance, EMT, angiogenesis, vasculogenic mimicry, invasion, migration, metastasis, metabolic reprogramming, and tumor microenvironment remodeling in BC. (a) Genes involved in cell proliferation, (b) cell death, (c) cell stemness, (d) metabolic reprogramming and tumor microenvironment remodeling, (e) angiogenesis and vasculogenic mimicry, and (f) invasion, migration, and metastasis.

Proliferation plays a crucial role in the advancement of BC, as it does in all types of cancers. Uncontrolled cell proliferation is a defining hallmark of cancer and contributes to the growth, invasion, and metastasis of tumors [52]. miR-204 has an important role in inhibiting proliferation in different cell types [53]. Target genes of miR-204 involved in proliferation in BC are shown in Figure 2. miR-204 downregulation in various types of cancer is related to an increase in cellular division and instauration of tumors [29,54,55]. For instance, it was found that overexpression of miR-204 in MCF7 BC cells inhibited their proliferation. The results showed that the upregulation of miR-204 triggered apoptotic cell death in MCF7 BC cells. Moreover, the analysis of the cell cycle revealed that miR-204 overexpression caused G2/M cell cycle arrest in these cancer cells. Also, it was shown that PTEN is a target of miR-204. Since PTEN regulates the PI3K/AKT signaling pathway, the effect of miR-204 overexpression was also assessed on this pathway and showed that miR-204 overexpression inhibits the expression of p-AKT and p-PI3K significantly in MCF7 [36] and MDA-MB-231 [23] BC cells. In the same way, T3 stimulation greatly enhanced the proliferation of MCF7 and T47D cells by decreasing the levels of miR-204, leading to increased expression of AREG, which in turn activates the AKT signaling pathway and promotes cell proliferation [56].

miR-204 might also have an anti-oncogenic impact on BC cells by suppressing the TGFβ pathway, since restoring miR-204 expression using RNA mimics in MDA-MB-231 and MCF-7 cells led to decreased cell proliferation [50]. Transcriptome analysis of MDA-MB-231 showed reduced expression of genes related to cell proliferation, such as ANGPT1 and TGβR2. Knocking down TGFβR2, but not ANGPT1, slowed down cell proliferation [37].

In addition, it has been shown that miR-204 directly targets FOXA1, binding to a complementary region. As a result, miR-204 controls the biological activities of BC cells by influencing cell proliferation through its interaction with FOXA1 [51]. These findings indicate that miR-204 acts as a tumor suppressor in BC by hindering proliferation and inducing apoptosis in MCF7 cells. In the same manner, miR-204 targets and negatively regulates the function of COX5A, indicating that by targeting and downregulating COX5A, miR-204 could inhibit the proliferation of MCF7 and T-47D cells in BC [57].

The modulation of miR-204 can be caused by the action of lncRNAs such as DSCAM-AS1, which is upregulated in BC tissue samples, while mir-204 is downregulated. DSCAM-AS1 was discovered to be increased in HCC1937 BC cells, leading to enhanced cell proliferation and apoptosis evasion by blocking miR-204-5p and raising RRM2 expression [58].

Conversely, the proliferation of MCF-7 and MDA-MB-231 cells is induced by miR-204 and miR-211, as assessed by cell proliferation and colony-forming assays, showing that tumor suppressors MX1 and TXNIP are direct targets of these miRNAs. Moreover, a strong association between miR-204 and miR-211 expression in BC tissue was observed. These results suggest that miR-204/211 contribute to increased cell proliferation, particularly in MCF-7 and MDA-MB-231 BC cells, through the downregulation of tumor suppressor genes [50].

### 3.2. Cell Death Resistance

Programmed cell death constitutes a natural mechanism that acts as a barrier against cancer development. Apoptosis, a type of programmed cell death, is triggered by the imbalance between pro- and anti-apoptotic molecules. This process involves the activation of caspases, which induce cell disintegration. Consequently, resistance to programmed cell death emerges as one of the most significant abilities that malignant cells acquire during oncogenesis, thereby facilitating successful tumor formation [24,52].

The balance between pro- and anti-apoptotic molecules can be modulated by intracellular and extracellular signals. Various miRNAs have been identified as regulators of programmed cell death. The role of miR-204 in BC is subject to controversy, as it has been associated with both anti-tumor and pro-tumor effects, due to its influence on modulating various aspects of cancer, particularly in promoting or resisting tumor cell death [50]. Target genes of miR-204 involved in cell death resistance in BC are shown in Figure 2.

On one hand, it is reported that miR-204 promotes the death of BC cells by regulating target genes involved in cell survival. Both cell lines and BC tissue samples tend to exhibit decreased expression of miR-204 [36,51,59]. This reduction has been related to a worse prognosis [29]. Interestingly, increased expression of miR-204 inhibits cell proliferation, leads to cell cycle arrest in the G2/M phase, and increases apoptosis [23,36,51], an effect that has been reversed when using miR-204 inhibitors [59].

Mechanistically, FOXA1 has been identified as a target of miR-204 [51]. FOXA1 is a transcription factor that promotes cell growth and inhibits apoptosis [60]. Therefore, by inhibiting FOXA1, miR-204 acts as a tumor suppressor promoting cell death [51]. Additionally, miR-204 downregulates Bcl-2 [59], and Bcl-2 specifically binds to FOXA1 [61], suggesting the existence of a positive feedback loop between miR-204, FOXA1, and Bcl-2, which together could modulate apoptosis through the STAT3/Bcl-2/survivin pathway [51].

A study conducted with BC tissue samples and cell lines revealed that another target gene of miR-204 is JAK2, whose signaling is associated with proliferation and apoptosis in cancer [62,63]. STAT3 is the main downstream target of phosphorylated JAK2 and is constitutively activated in various tumors, exerting oncogenic and anti-apoptotic functions [64]. When STAT3 dimerizes and translocates to the nucleus, it regulates the transcription of anti-apoptotic genes Bcl-2 and survivin [65,66]. Therefore, when miR-204 suppresses JAK2, it inhibits the activation of STAT3, Bcl-2, and survivin [59].

Consistent with reports supporting the role of miR-204 in inducing cancer cell death, a research group used bioinformatics tools to identify PTEN as a target of miR-204. However, in this case, the overexpression of miR-204 also induces an increase in PTEN expression. PTEN is the major brake of the PI3K/Akt signaling pathway [67]; therefore, the overexpression of miR-204 significantly reduces p-Akt and p-PI3K, affecting cell survival [36]. Complementarily, it has also been reported that PIK3CB, the main regulator of the PI3K/Akt pathway, is a direct target of miR-204-5p, the major strand of mature miR-204 [20]. Accordingly, the overexpression of miR-204-5p limits the viability of BC cells by decreasing the expression of PI3KCB [23].

Although apoptosis was considered the main form of programmed cell death some years ago, it is now recognized that cell death can occur through various programmed mechanisms, many of which have been identified to be involved in carcinogenesis [68]. Recently, it has been described that intracellular copper accumulation contributes to the aggregation of mitochondrial lipoylated proteins, triggering a novel form of programmed cell death called cuproptosis [69]. However, there is evidence that deregulation in copper levels promotes the development and progression of cancer, although more studies on this type of cell death are needed [70]. An analysis conducted with online database information identified that overexpression of miR-204-5p in BC was associated with better overall survival. Additionally, it has been proposed that miR-204-5p could be an upstream regulator of SLC31A1, a gene associated with cuproptosis in BC [71].

Contrary to findings suggesting that miR-204 induces programmed cell death in cancer cells, certain research has linked increased miR-204 expression with resistance to tumor cell death. A study conducted with BC cell lines and tissue samples identified tumor suppressors MX1 and TXNIP as targets of miR-204, suggesting that the functions of this miRNA are directed toward cell death suppression and proliferation stimulation [50].

In the same context, with in vitro and in vivo models of estrogen receptor (ER)-negative and ER-positive BC, downregulation of miR-204 has been observed to promote ERα expression. Consequently, it has been proposed that miR-204 regulates ERα [72], an important regulatory axis since ERs play a crucial role in BC evolution and treatment response. Loss of these receptors is associated with increased tumor aggressiveness and worse prognosis [73]. Notably, miR-204 is considered to affect Akt phosphorylation by ERα [74]. It was observed that when miR-204 decreased or ERα increased, Akt phosphorylation decreased, exerting an inhibitory effect on Mcl-1 expression. Since Mcl-1 contributes to regulating the balance between cell survival and death signals, tumor cells can increase their expression to avoid apoptosis and proliferate uncontrollably [72].

Additionally, it has been demonstrated that miR-204 significantly inhibits caspase-3 activity after Tamoxifen treatment [72], a chemotherapy drug that inhibits ERα target gene expression, regulating the cell cycle and apoptosis [75]. Conversely, decreased miR-204 expression increases the sensitivity of BC cells to Tamoxifen treatment and enhances caspase-3 activity [72]. Consequently, miR-204 is implicated in resistance to cell death directly and in response to chemotherapy.

Taken together, it has been experimentally demonstrated that miR-204 can exert a dual effect on BC cells, both inducing and inhibiting programmed cell death. As previously proposed by some authors [50], these divergent findings could indicate that miR-204 may play a dual role in BC development and progression, where the anti-tumorigenic or pro-tumorigenic effect could depend on the specific type and status of the tumor.

### 3.3. Epithelial–Mesenchymal Transition

EMT in BC is a biological process that results in increased invasiveness and metastatic potential. miR-204 has been found to play a crucial role in regulating EMT in BC [76]. Several studies have demonstrated that miR-204 acts as a tumor suppressor in BC by targeting key molecules involved in the EMT process (Figure 2); for instance, miR-204 has been shown to directly target and inhibit ZEB2, a transcription factor that promotes EMT, and it is also suggested that MALAT1 might enhance the EMT phenotype via the miR-204/ZEB2 axis. According to this proposal, MALAT1 acts as an endogenous sponge to negatively regulate miR-204 expression. Subsequently, miR-204 suppresses ZEB2 expression by binding to the non-coding region of ZEB2 3′-UTR [77]. Therefore, MALAT1 regulates the miR-204/ZEB2 axis in BC. Also, miR-204 directly targets the gene Six1, which is upregulated in BC specimens. Overexpression of Six1 leads to the downregulation of miR-204, which contributes to the promotion of EMT in BC. This regulatory circuit between miR-204 and Six1 constitutes a feedback loop that influences the progression of EMT in BC cells [78]. In BC cell lines, it has been also reported that the lncRNA ARNILA can sequester miR-204, acting as a competing endogenous RNA (ceRNA) and promoting EMT by competitively binding to miR-204 while upregulating Sox4 [79].

In an integrative analysis, the expression patterns of several miRNAs related to processes linked to metastasis were evaluated. Dysregulation was observed in miR-204, miR-200c, miR-34a, and miR-10b, potentially resulting in a reduced survival rate in BC. Furthermore, these miRNAs could be modulated through the overexpression of OCT4, SOX2, KLF4, c-MYC, NOTCH1, SNAI1, ZEB1, and CDH2, genes directly associated with EMT [80].

### 3.4. Stemness

Cancer stem cells (CSCs) are transformed cells with self-renewal capacity, the ability to initiate tumor formation, the capability to disseminate to distant sites, clonal long-term repopulation potential, and phenotypic plasticity to differentiate into other cell types, both stem and non-stem states [81,82]. Considering that stemness involves self-renewal and differentiation capacity in tumor cells, vital processes in carcinogenesis, it has been proposed as a hallmark of cancer [83].

The recurrence of BC in patients treated with chemotherapy or surgical interventions is a significant challenge in managing this disease [84]. BC stem cells (BCSCs) are believed to be primarily responsible for this phenomenon because, due to their potential for self-renewal and differentiation in multiple directions, they act as seed cells producing new malignant cells [85]. Surprisingly, most deaths associated with this type of cancer are not attributable to the primary tumor but to metastasis to other organs [86], a process closely associated with the presence and activity of BCSCs. Additionally, these cells exhibit unique resistance to chemotherapy, underlining their crucial role in BC progression and recurrence [87].

Stemness is a process that can be post-transcriptionally regulated by miRNAs. In this regard, it has been reported that miR-204 may promote the presence of BCSCs (target genes of miR-204 involved in cell stemness in BC are shown in Figure 2). A study conducted bioinformatics analysis and in vitro assays to analyze this hallmark in BC and identified miR-204 targets including CD44, FOXC1, HOTTIP, MYC, NOTCH1, SOX2, STAT3, and VIM1, genes involved in pathways associated with self-renewal [80].

Conversely, another study has suggested that miR-204 may suppress stemness. One target of miR-204 is SAM68, a molecule that positively correlates with the self-renewal potential of BC cells by activating the Wnt/β-catenin pathway [85]. Consistently, a study with tumor specimens, cell lines, and a murine model of BC reported that miR-204 can bind to the 3′-UTR of TCF4. The downregulation of miR-204 then upregulates TCF4, leading to the activation of the Wnt/β-catenin signaling pathway [87]. This effect is explained given that TCF4, along with the coactivator β-catenin, functions as the main transcriptional mediators of the Wnt pathway [88]. It is important to note that aberrant activation of the Wnt/β-catenin signaling pathway plays a crucial role in the origin and maintenance of CSCs [89].

These findings highlight that the role of miR-204 in inducing CSCs remains controversial [80]. Most studies consider miR-204 as a tumor suppressor; accordingly, its reduced expression is associated with a more aggressive phenotype of BC [90]. Nevertheless, some research suggests that miR-204 could be an oncomiR in BC [91].

The plasticity of CSCs is particularly important in cancer evolution, allowing them to adapt and survive the stressful conditions of the tumor microenvironment (TME), even in the face of alterations induced by oncologic therapy. Therefore, CSCs can also induce treatment resistance by promoting metabolic reprogramming in the TME [81].

### 3.5. Metabolic Reprogramming

The basis of malignant neoplasia lies in the capacity for uncontrolled proliferation and migration acquired by tumor cells, which requires adjustments in energy metabolism to drive this process. This metabolic reprogramming is orchestrated mainly by proteins involved in other cancer hallmarks [92], leading to an understanding of how the integration of all these distinctive characteristics acquired by cancer cells allows for cancer development and progression.

This metabolic reprogramming involves energy changes in both tumor and non-tumor cells, implying complex pathways of cellular regulation as well as intercellular communication, in which miRNAs may participate. Through studies with cell cultures and murine models, it has been identified that the overexpression of miR-204 alters genes involved in the metabolism of BC cells, achieving a significant metabolic suppression in them (target genes of miR-204 involved in metabolic reprogramming in BC are shown in Figure 2) [23].

Additionally, it has also been reported that the presence of miR-204 in blood is associated with hypermetabolism and energy consumption in BC patients, since miR-204-5p secreted in exosomes by tumor cells targets the VHL gene in adipose tissue, inducing the expression of the HIF1A protein and, in turn, the activation of the leptin signaling pathway [93]. Leptin derived from adipocytes regulates the expression of genes associated with tumor progression, such as those involved in adhesion, invasion, angiogenesis, and apoptosis [94,95]. Furthermore, leptin signaling increases lipolysis in white adipose tissue, promoting cachexia [93], a multiorgan wasting syndrome characterized by systemic inflammation accompanied by the loss of adipose tissue and skeletal muscle [96]. Therefore, increased leptin signaling in the body is associated with BC aggressiveness [93].

It is crucial to consider that changes in metabolism also involve the metabolic reprogramming of non-tumor cells present in the TME, which is the result of an adaptation process to factors derived from tumor cells. It has been reported that BC cells secrete extracellular vesicles containing miR-204. These vesicles are subsequently taken up by nearby cancer-associated fibroblasts (CAFs), where miR-204 acts on RAGC, a component of Rag GTPases that regulates mTORC1 signaling. mTORC1 is a protein kinase that coordinates cell growth in response to available nutrients. Consequently, by educating CAFs to reduce mRNA translation, BC cells remodel the amino acid metabolic flux and regulate proteins produced by the stroma during periods of nutrient fluctuation [97]. As a result of this suppression in protein synthesis, CAFs could utilize intracellular amino acids to produce energy and possibly transfer energy to cancer cells through the secretion of metabolites. Additionally, suppression of mTORC1 signaling induces an increase in the synthesis of certain proteins associated with autophagy, lipid metabolism, survival, and intercellular communication, contributing to the survival of CAFs to continue favoring the growth of tumor cells [97]. In this way, CAFs participate in shaping tumor metabolism by providing nutrients to cancer cells and modulating their metabolic pattern [98,99], highlighting the importance of the interaction between TME cell populations to sustain this and other cancer hallmarks.

### 3.6. Tumor Microenvironment Remodeling

Cancer involves the formation of a complex ecosystem called the TME, where tumor cells interact with other non-cancerous cell populations, collectively driving tumor formation, progression, and treatment response [100,101]. The TME promotes a state of chronic inflammation that contributes to multiple hallmark capabilities by providing bioactive molecules such as growth factors that sustain tumor proliferation, survival factors that limit cell death, matrix remodeling enzymes that facilitate invasion and metastasis, as well as cytokines that modulate various cellular programs [92].

The interaction between tumor cells and their microenvironment is a crucial determinant of the abilities acquired by cancer [102]. Therefore, intercellular communication in the TME is of great relevance and involves complex networks in which molecules such as miRNAs can participate. Models of BC in vivo show that miR-204 shapes the TME by regulating the expression of key cytokines involved in monocyte and lymphocyte infiltration (target genes of miR-204 involved in tumor microenvironment modeling in BC are shown in Figure 2) [23].

Tumors with a higher expression of miR-204 show reduced infiltration of CD11b+ myeloid cells and fewer myeloid-derived suppressor cells (MDSCs), macrophages, and NK cells. Nevertheless, the increase in miR-204 expression is associated with an enhancement in infiltrating CD8+ and CD4+ T cells, including regulatory T cells [23]. This phenomenon is related to the fact that the overexpression of miR-204 induces dysregulation in cytokine production [23]. This modulation of immune cell infiltration is relevant, as the reduction in myeloid cell chemotaxis in the TME may be associated with decreased metastasis, as myeloid cells tend to promote tumor invasion and migration [103].

Regarding this dysregulation in cytokine production in the TME, it has been identified that miR-204 induces a decrease in the expression of genes involved in TGF-β signaling, including PTGS2, which is implicated in the expression of IL-11. Additionally, miR-204 directly binds to the IL11 3′-UTR, exacerbating the reduction in IL-11 production by BC cells [104]. Complementarily, miR-204 has been observed to inhibit the lncRNA DGUOK-AS1, a molecule that promotes cell migration, angiogenesis, and macrophage migration by inducing an increase in IL-11 production [105].

IL-11 is an IL-6 family cytokine that can be secreted by various cells in the TME in response to inflammatory stimuli [106,107]. In this regard, IL-11 plays an important role in promoting angiogenesis, invasion, and migration of tumor cells, as well as in proinflammation and differentiation of tumor-associated macrophages (TAMs) [108,109]. Cancer cells can recruit monocytes to the TME through the secretion of cytokines and chemokines [110], which subsequently differentiate into TAMs and, together with other cells in the microenvironment, modulate tumor behavior. Thus, TAM infiltration has been significantly associated with clinical behavior and chemoresistance in BC [105]. Consequently, high expression of IL-11 has been related to a high histological grade, poor survival [111], and BC bone metastasis development [112].

Interestingly, miR-204 not only shapes the TME through the regulation of cytokine production but also by modulating the expression of immune checkpoints. It is reported that miR-204 is a potential upstream regulator of SLC31A1, which is positively correlated with the expression of immune checkpoints, notably CD274 and CTLA4, regulating the effective response of T lymphocytes. Therefore, the expression of miR-204 could also impact the efficacy of immunotherapy [71]. The available information highlights the importance of miR-204 in shaping the TME in BC, which impacts tumor progression, treatment response, and consequently, the clinical evolution of patients. Regardless, additional studies are needed to evaluate the effect of this miRNA on the behavior of other populations present in the TME, as well as the effect of factors produced by non-tumor cells on the expression of miR-204 in BC cells.

### 3.7. Angiogenesis and Vasculogenic Mimicry

In a normal physiological context, angiogenesis plays a vital role in facilitating the growth of new blood vessels from pre-existing ones. This process is essential for the transportation of oxygen and nutrients, thereby ensuring the proper functioning of tissues and organs [113]. However, in a tumor context, the microenvironment is primarily composed of endothelial cells, which play a significant role in the formation of new blood vessels that not only supply oxygen and nutrients to the tumor but also facilitate the creation of conduits that direct blood flow, thereby maintaining tissue perfusion [113,114,115]. In the tumor microenvironment, there exists an imbalance between proangiogenic and antiangiogenic factors, which ultimately favors tumor progression [113,114,115].

Several miRNAs function as either oncogenes or tumor suppressors, modulating molecules involved in angiogenesis, a critical process for tumor progression. These miRNAs are referred to as angiomiRs [116], as they regulate angiogenic mechanisms in both normal physiological and pathological contexts. Recently, miR-204 has been related to this process, modulating the expression of key molecules (target genes of miR-204 involved in angiogenesis and VM in BC are shown in Figure 2). For instance, in BC cell lines, where vasculogenic mimicry (VM) formation (in vitro model simulating the generation of three-dimensional channels facilitating oxygen and blood supply) was induced, transfection with miR-204 inhibited VM formation under hypoxic conditions. This inhibition led to a reduction of over 80% in the formation of branch points and capillary tubes compared to non-transfected cells. Additionally, the study evaluated hypoxia-inducible factor-1α (HIF-1α), one of the principal regulators of angiogenesis. A decrease in HIF-1α protein expression was observed in cells transfected with miR-204 [117]. In addition, it was reported that in cancer stem cells in CD44^+^- and CD29^−^-positive BC cell lines, under hypoxic conditions and following transfection with miR-204, vasculogenic mimicry (VM) formation was reduced, leading to a decrease in branch points. Additionally, a decrease in the protein expression of β-catenin and VEGFA was observed [118].

In addition, various models have demonstrated the significant role of miR-204 in tumor angiogenesis. Its downregulation has been identified in BC samples, cell lines, and in vivo models. An inverse correlation has been established between miR-204 expression and the miRNA-204/*ANGPT1/TGFβR2* axis, wherein lower miR-204 expression is associated with higher expression levels of ANGPT1 and TGFβR2 proteins [37].

Lastly, long non-coding RNAs have been discovered to act as sponges, sequestering specific miRNAs, such as HOTAIR. HOTAIR is upregulated in BC tumors and is believed to sequester miR-204. It has been identified that HOTAIR contains a conserved potential binding site for miR-204. Consequently, cell lines exhibiting high miR-204 expression demonstrated low expression levels of HOTAIR. Also, in a bioinformatics analysis, it was found that miR-204 can bind to the 3′-UTR region of focal adhesion kinase 1 (*FAK*), which has even been found to be related to processes of migration and vasculogenic mimicry [119].

### 3.8. Invasion, Migration, and Metastasis

Some of the fundamental processes for malignant progression are migration, invasion, and metastasis. These processes are essential for tumor cells to spread successfully. Migration depends on the morphology of the cells that will migrate, thus relying on various factors such as genetic and molecular cell–cell junctions, rearrangement of the cytoskeleton, and adhesion to the matrix [120,121]. On the other hand, invasion and metastasis can occur through a series of steps. This process starts with local invasion, followed by the intravasation of cancer cells into blood vessels. Subsequently, extravasation takes place, involving the migration of cancer cells to distant tissues. Then, micrometastasis develops, forming small nests of cancer cells. Finally, colonization occurs, as these tumor nests or micrometastatic lesions grow into tumors [92,122].

It has been shown that low expression of miR-204 enhances key processes such as migration, invasion, and metastasis. Therefore, in BC samples, the microdeletion of genomic loci specifically containing miR-204 has been directly linked to activation pathways in tumor progression [90]. In addition, transfection with miR-204 inhibits migration and invasion. Additionally, a bioinformatics analysis revealed that the *PTEN* gene is a target of miR-204, which consequently negatively modulates signaling pathways such as PI3K/AKT. As a result, miR-204 inhibits metastasis in BC cell lines by targeting the PI3K/AKT signaling pathway [36].

Downregulation of miR-204 has been observed in cell lines enhancing overexpression of *FOXA1*. Consequently, increasing miR-204 expression leads to the suppression of cell invasion and metastasis processes through FOXA1 downregulation [51]. Another target of these miRNAs is the adaptor protein complex 1, sigma subunit 3 (*AP1S3*), which is also overexpressed in BC. However, upregulation of miR-204 significantly block cancer cell migration and invasion by downregulating *AP1S3* at the protein level [123]. Also, the role of miR-204 in regulating a member of the atypical right open reading frame (RIO) protein kinase family, *RIOK1*, was investigated. It was observed that there was a significant reduction in migration in in vitro assays, thereby impacting tumor progression [124].

In addition, significant overexpression of the long non-coding RNA DSCAM-AS1 (Down syndrome antisense cell adhesion molecule) has been observed in BC samples and a specific cell line. This lncRNA functions as an endogenous competitor (ceRNA) of miR-204. A negative association between DSCAM-AS1 and miR-204 has been shown to enhance BC cell migration. However, when co-transfection of DSCAM-AS1 and miR-204 is carried out, the *RRM2* Ribonucleotide reductase M2 (*RRM2)* gene is suppressed in BC cells; thus, DSCAM-AS1/miR-204-5p/*RRM2* is an important pathway in BC to support cell migration and invasion is observed [58].

It has been observed that high expression of exosomal circRHOT1 leads to downregulation of miR-204 expression and the gene that regulate this miRNA is Protein arginine methyltransferase 5 (*PRMT5)*, so the miR-204-5p/*PRMT5* axis could participate in cancer progression. However, when circRHOT1 is suppressed, miR-204 expression is significantly increased. In the same study, an in vivo model was utilized, where a miR-204 inhibitor was combined with circRHOT1 suppression. This combination facilitated the recovery of cell function and promoted migration, invasion, and other processes related to tumor progression. These effects were achieved by increasing the expression of E-cadherin and N-cadherin while decreasing vimentin, thus inhibiting the EMT process [125].

## 4. Bullet Points of the Role of miR-204 in the Hallmarks of Breast Cancer

In summary, miR-204 is implicated in the instauration of fundamental traits acquired during the multistep development of BC (Figure 2). Furthermore, the molecules regulated by miR-204 form associated networks across various hallmarks of cancer, as revealed by STRING analyses. This underscores the versatility of this microRNA in regulating diverse cellular processes that are relevant for BC development [126] (Supplementary Figure S1 and Supplementary Table S1).

- Cell proliferation. miR-204 seems to have a dual role in BC proliferation. While it appears to primarily function as a tumor suppressor by inhibiting proliferation and inducing apoptosis, some studies suggest that it can also promote proliferation in specific contexts. For instance, miR-204 overexpression inhibits proliferation in MCF7 BC cells by triggering apoptosis and causing G2/M cell cycle arrest. It also suppresses the TGFβ pathway and targets FOXA1, further contributing to its anti-proliferative effects. However, miR-204, alongside miR-211, has been shown to induce proliferation in MCF-7 and MDA-MB-231 cells by downregulating tumor suppressor genes MX1 and TXNIP. This contrasting effect highlights the complexity of miR-204’s role in BC. Therefore, further research is needed to fully elucidate the context-dependent roles of miR-204 in BC proliferation. Understanding these intricacies is crucial for developing targeted therapies that effectively manipulate miR-204 activity for therapeutic benefit.
- Cell death resistance. miR-204 seems to have a complex and multifaceted role in BC cell death resistance. While some studies suggest that it promotes cell death by regulating genes like FOXA1, Bcl-2, JAK2, and PTEN, others indicate that it might suppress cell death by targeting tumor suppressors like MX1 and TXNIP and influencing ERα signaling. Therefore, it is difficult to definitively conclude whether miR-204 has a net pro-tumor or anti-tumor effect in BC. Its role may be context-dependent, varying based on factors like the specific type and stage of the tumor, the presence of other mutations, and the tumor microenvironment. Further research is needed to fully elucidate the mechanisms of action of miR-204 in BC and determine its potential as a therapeutic target.
- EMT. miR-204 plays a crucial role in regulating EMT in BC. It acts as a tumor suppressor by targeting key molecules involved in the EMT process, such as ZEB2 and Six1. Downregulation of miR-204, potentially influenced by molecules like MALAT1, can lead to increased expression of these EMT-promoting factors, thereby enhancing the EMT phenotype and promoting invasiveness and metastasis in BC. Conversely, upregulation of miR-204 could potentially inhibit EMT and suppress tumor progression.
- Stemness. The role of miR-204 in breast cancer stemness is complex and appears to be context-dependent. While some studies suggest that miR-204 may promote stemness by targeting genes involved in self-renewal pathways, others indicate that it could suppress stemness by inhibiting the Wnt/β-catenin signaling pathway through targeting SAM68 and TCF4. Further research is needed to fully elucidate the precise role of miR-204 in BC stemness and its implications for therapeutic targeting.
- Metabolic reprogramming. miR-204 acts as a key regulator of metabolic reprogramming in breast cancer by directly suppressing metabolic genes within tumor cells, leading to decreased metabolic activity and potentially limiting energy production for tumor growth; indirectly affecting metabolism through exosome signaling: tumor-secreted miR-204 targets VHL in adipose tissue, activating HIF1A and the leptin pathway, and this results in increased lipolysis, cachexia, and tumor progression; and remodeling metabolic flux within the tumor microenvironment: miR-204-containing exosomes are taken up by cancer-associated fibroblasts, suppressing mTORC1 signaling. This limits CAF protein synthesis, potentially diverting resources to fuel tumor cells and promoting tumor survival.
- Tumor microenvironment modeling. miR-204 orchestrates a complex interplay within the breast cancer TME by reducing infiltration of pro-tumorigenic myeloid cells while enhancing infiltration of anti-tumorigenic T cells, potentially limiting tumor invasion and enhancing immune surveillance; it also suppresses pro-tumorigenic IL-11 production, avoiding angiogenesis, tumor cell migration, and macrophage differentiation. Finally, as a regulator of SLC31A1, which correlates with immune checkpoint expression, miR-204 might impact the efficacy of immunotherapies targeting these checkpoints.
- Angiogenesis and vasculogenic mimicry. miR-204 inhibits angiogenesis and vasculogenic mimicry in breast cancer by reducing the VM formation of branch points and capillary tubes in BC cell lines, disrupting the development of these vascular channels. miR-204 suppresses the expression of HIF-1α, a crucial regulator of angiogenesis, and reduces the protein expression of β-catenin and VEGFA, both involved in VM formation. HOTAIR, a lncRNA upregulated in BC, is believed to sequester miR-204; this suggests a potential competitive mechanism where HOTAIR upregulation could promote angiogenesis by limiting miR-204’s inhibitory effects. miR-204 binds to the 3′-UTR region of FAK, a protein linked to migration and VM formation, potentially hindering these processes.
- Invasion, Migration and Metastasis. miR-204 inhibits breast cancer progression by targeting multiple genes involved in tumor progression such as FOXA1, AP1S3, RIOK1, RRM2, and PRMT5. It negatively regulates the PI3K/AKT pathway by targeting PTEN, further inhibiting metastasis. miR-204 can be sequestered by lncRNAs like DSCAM-AS1, which promotes cell migration. Also, suppressing circRHOT1, miR-204 indirectly increases E-cadherin and N-cadherin expression while decreasing vimentin, ultimately inhibiting EMT.

## 5. Clinical Relevance of miR-204 in Breast Cancer

miR-204 regulates chemotherapy resistance in BC by targeting specific genes involved in tumor growth and chemotherapy drug reactivity. In the context of estrogen receptor-positive (ER^+^) BC, miR-204 can bind to the 3′-UTR of the gene COX5A, inhibiting its expression, leading to reduced viability and invasiveness of BC cells, and sensitizing them to chemotherapy drugs like Doxorubicin (DOX). When miR-204 is introduced to ER^+^ BC cells, it not only diminishes their growth and invasion abilities but also enhances the effectiveness of DOX in reducing cell viability. These effects can be reversed by the overexpression of COX5A, suggesting that miR-204’s regulatory effect on chemotherapy resistance operates through its interaction with COX5A [57].

Moreover, studies have shown that the manipulation of miR-204 levels can impact the effectiveness of other BC treatments. For instance, Trichostatin A, a drug that influences miR-204 expression, can increase the expression of ERα and, alongside Tamoxifen (TAM), enhance cancer cell sensitivity to treatment by reducing the activity of pathways such as AKT/mTOR, which are involved in cancer cell survival. When miR-204 expression is reduced, the inhibition of BC cells by TAM is enhanced, indicating that miR-204’s modulation may be a potential strategy to overcome treatment resistance in BC [72]. In summary, miR-204’s regulation of genes such as COX5A and ERα plays a crucial role in influencing the chemoresistance of ER^+^ BC cells, making it a potential target for improving the effectiveness of chemotherapy in BC treatment.

Today, nanotechnology emerges as a promising therapeutic avenue. The synergistic application of gold nanoparticles (AuNPs) and miRNAs holds potential as an effective therapeutic strategy. Studies have indicated that the combined utilization of AuNPs and miR-204 overexpression contributes to the regulation of the MMP9 protein, pivotal in invasion and metastasis processes. This approach suppresses MMP9 expression via the NF-κB signaling pathway [127].

Moreover, miR-204 is identified as an angiomiR and holds promise as a crucial therapeutic target in angiogenesis regulation. In vivo nude mouse models employing angioreactor technology revealed a substantial impact of miR-204 on blood vessel formation. These findings underscore the potential of miR-204 as a significant therapeutic target in breast cancer treatment, thereby unveiling new avenues in preclinical research [37].

Importantly, the expression of miR-204 has been linked to the clinical behavior, treatment response, and prognosis of BC [29,71,72,90] (Table 1). miR-204 has been identified as a regulator of several cancer hallmarks, suggesting that its expression could serve as a biomarker for tumorigenesis and BC prognosis [59,128], even by blood assessment [80].

Additionally, given miR-204’s ability to modulate each distinctive characteristic of cancer, it has been suggested that manipulating its expression could be a potential therapeutic target in BC. For instance, it could be employed to modulate cancer cell death [50,51,59], eliminate BCSCs [85], reprogram metabolism, shape the TME, and enhance response to immunotherapy [71].

Nevertheless, it has been observed that miR-204 may play a dual role in directing various BC hallmarks [23], highlighting the need for further studies to better understand its role in orchestrating BC behavior. This would allow proposing miR-204 as a potential diagnostic marker, prognostic indicator, or therapeutic target with greater precision.

## 6. Conclusions

The exploration of the role of miR-204 in the hallmarks of BC reveals a complex landscape of interactions influencing various aspects of tumor development and progression. This microRNA emerges as a potent tumor suppressor, exerting its regulatory influence on processes such as angiogenesis, vasculogenic mimicry, invasion, migration, and metastasis. Its intricate interplay with diverse signaling pathways, coupled with its ability to modulate the tumor microenvironment, highlights its significance in breast cancer pathogenesis. However, the complex nature of miR-204 requires further investigation.

Despite its promising potential as a therapeutic target and prognostic marker, a deeper understanding of its context-dependent roles and interactions within the intricate tumor ecosystem is crucial. Elucidating the full scope of miR-204’s influence will pave the way for innovative therapeutic strategies and personalized treatment approaches for breast cancer patients.

## Figures and Tables

**Table 1:** miR-204’s effect on clinical behavior, treatment response, and prognosis in breast cancer patients.

Study	Number of Participants	Purpose	Results	Reference
Decreased expression of miR-204 is associated with poor prognosis in patients with breast cancer.	A total of 129 female BC patients were included, from whom tumor tissues were obtained.	To examine clinical significance of miR-204 expression in tissues from BC patients.	MiR-204 expression was significantly associated with TNM stage, metastasis, and chemotherapeutic resistance in BC patients. Patients with low miR-204 expression had poorer overall survival and disease-free survival. The study concludes that miR-204 may be a potential diagnostic and prognostic biomarker for BC.	[29]
Identification of cuproptosis-related gene SLC31A1 and upstream LncRNA-miRNA regulatory axis in breast cancer.	Data from 1076 female BC patients were analyzed, with information downloaded from the TCGA database. Additionally, for PCR validation, four pairs of BC samples and normal breast tissues were collected.	To conduct a comprehensive analysis of the regulatory network of cuproptosis-related genes in the context of BC.	SLC31A1 is a cuproptosis-related gene in BC; its expression is increased in cancer samples and it is negatively correlated with favorable outcomes. miR-204 is an upstream regulator of SLC31A1. The study concludes that the LINC01640/miR-204-5p/SLC31A1 axis might be significant and promising in the context of cuproptosis in BC.	[71]
Trichostatin A and tamoxifen inhibit breast cancer cell growth by miR-204 and ERa suppressing AKT/mTOR pathway.	Female nude mice (6–8 weeks old) received a subcutaneous injection with MCF-7 cells and MDA-MB-231 cells.	To investigate miR-204 function in breast cancer, using Trichostatin A (TSA) to treat breast cancer cell lines MCF-7 (ER^+^) and MDA-MB-231 (ER^−^).	TSA and TAM combination inhibits Mcl-1 expression by decreasing phosphorylation of AKT induced by ERα increase in vivo and in vitro. Also, TSA could upregulate ERa by increasing miR-204 in MDA-MB-231 cells.	[72]
Genomic loss of miRNA-204 promotes cancer cell migration and invasion by activating AKT/mTOR/Rac1 signaling and actin reorganization.	Fifteen breast cancer tissues, the cell line of breast cancer (MDA-MB-231), and samples from other types of cancer.	Analysis of genomic loci encoding miR-204 in multiple cancer types, including ovarian, pediatric renal tumors, and breast cancers.	Results reveal that chromosomal loci containing miR-204 are frequently lost, resulting in its lower expression in multiple cancers. miR-204 acts as a tumor growth and metastasis suppressor.	[90]

## Supplementary Materials

The following supporting information can be downloaded at: https://www.mdpi.com/article/10.3390/cancers16162814/s1, Figure S1. The molecules regulated by miR-204 form associated networks across various hallmarks of cancer, as revealed by STRING analyses; Table S1. String links of every hallmark regulated by miR-204.
