# Supplementary material for: Role of MicroRNA-204 in Regulating the Hallmarks of Breast Cancer: An Update
Source: Cancers (Basel). 2024 Aug 10;16(16):2814. doi: 10.3390/cancers16162814 (PMC11352763; doi:10.3390/cancers16162814)
Supplement: Supplementary file 1 [file cancers-16-02814-s001.zip › Supplementary Table 1.pdf]

Supplementary Table 1. String links of every hallmark regulated by miR-204.

**Hallmark regulated by miR-204**	**Link to String**
Cell proliferation	https://string-db.org/cgi/network?taskId=bkfKd1yITvhB&sessionId=bp1Pp2iWnzqh
Cell death resistance	https://string-db.org/cgi/network?taskId=bzAybydlbJRg&sessionId=boUk7eOnNvyo
Epithelial-Mesenchymal Transition	https://string-db.org/cgi/network?taskId=b0Dcfe0AXQjd&sessionId=bse6MnZ3MkVA
Cell stemness	https://string-db.org/cgi/network?taskId=bHhrAugVGRBZ&sessionId=bjh8Z32rgdHL
Metabolic reprogramming and tumor microenvironment remodeling	https://string-db.org/cgi/network?taskId=bmN3rbdNkjNc&sessionId=bse6MnZ3MkVA
Angiogenesis and vasculogenic mimicry	https://string-db.org/cgi/network?taskId=bbbm7b6OPxlP&sessionId=boUk7eOnNvyo
Invasion, migration and metastasis	https://string-db.org/cgi/network?taskId=bo5aE5spEkyE&sessionId=bse6MnZ3MkVA
