# Supplementary figures and images for: Role of MicroRNA-204 in Regulating the Hallmarks of Breast Cancer: An Update
Source: Cancers (Basel). 2024 Aug 10;16(16):2814. doi: 10.3390/cancers16162814 (PMC11352763; doi:10.3390/cancers16162814)

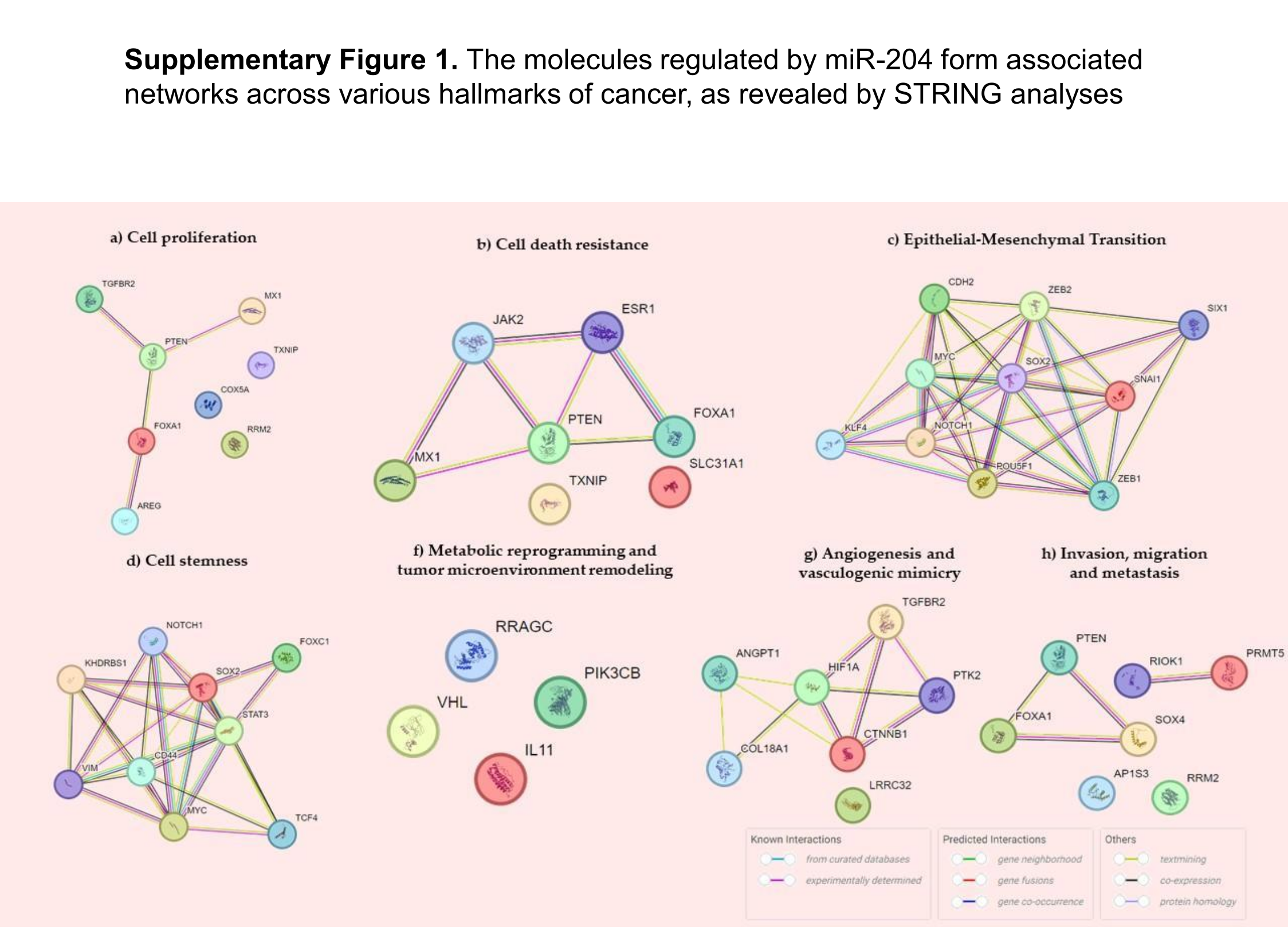

Supplement: Supplementary file 1 [file cancers-16-02814-s001.zip › Supplementary Figure 1.tif]
